# Insight into electrochemical degradation of Cartap (in Padan 95SP) by boron-doped diamond electrode: kinetic and effect of water matrices

**DOI:** 10.55730/1300-0527.3476

**Published:** 2022-05-11

**Authors:** Nguyen Tien HOANG

**Affiliations:** The University of Da Nang, University of Science and Education, Da Nang, Vietnam

**Keywords:** BDD electrode, Cartap degradation, mechanism, kinetic, cyclic voltammetry, linear sweep voltammetry

## Abstract

In this work, the kinetic electrochemical degradation of Cartap (CT) (in Padan 95 SP) at boron-doped diamond (BDD) electrode was investigated. This study indicated that the degradation of CT underwent both direct and indirect oxidations. Water matrices can either accelerate or inhibit the removal efficiency of CT: adding 15 mM Cl^−^ improved *k_CT_* from 0.039 min^−1^ to 0.054 min^−1^ (increased by 38%), while *k_CT_* decreased by 61.5% and 64% when increasing the concentration of HCO_3_^−^ and humic acid (HA) to 15 mM and 15 mg L^−1^, respectively. CT degradation was inhibited in the presence of methanol (MeOH) and *tert*-butanol (TBA) due to the scavenging effect of those chemicals toward reactive species. The contribution of reactive oxidants was calculated as: DET (direct electron transfer) accounted for 15%; •OH accounted for 61.5%; SO_4_^•−^ accounted for 12.8%; ROS (the other reactive oxygen species) accounted for 8.5%. The transformation pathways of major reactive species were established.

## 1. Introduction

Pesticide is a toxic chemical substance, which usually contains more than two agents (including biological compounds) which are added into pesticide to control fungal or animal pests. However, due to the uncontrolled applications or the overuse of pesticides, the environment was seriously polluted. Depending on the chemical property, some pesticides can exist in the environment with different lifetime (from several days to several month and even e few decades). In some areas, the concentration of pesticides in agricultural wastewater is up to 500 mg L^−1^ that causes the serious environmental pollutions [1,2].

The powerful treatment methods should be developed and applied to decompose these polluted chemicals. To solve this urgent issue, the advanced oxidation processes (AOPs) are considered as an effective method to successfully remove none-biological degraded compounds from water, for example, ozonation, Fenton/photo-Fenton and photocatalysis processes [3,4]. However, the above methods have some disadvantages: ozonation requires the complicated equipment with high operating and maintenance costs [5]; Fenton/photo-Fenton discharges the high concentration of anions in the treated solution and large amounts of sludge; the low removal efficiency of photocatalysis process can be attributed to the low adsorption possibility of organics on the TiO_2_ and the aggregation of TiO_2_ because of the instability of the nanosized particle [6]. The electrochemical oxidation (EO) process is a promising technique to treat wastewaters containing toxic, refractory organic pollutants [7]. In EO process there are two possible mechanism pathways that involve in organics degradation: (1) direct oxidation in which the organic is directly oxidized thanks to the electron transfer on the anode surface; (2) indirect oxidation where various oxidizing species (i.e. •OH, SO_4_^•−^, •OOH or H_2_O_2_, O_3_, singlet oxygen, ${\text{O}}_{2}^{•}$). In addition, other related electrolyte species also contribute to the organic decomposition [8]. The description of the interaction between oxidizing species and target compounds for both mechanism ways (direct and indirect oxidation) can be simplified in Eq. (1):


(1) $$\text{Organic}+{\text{e}}^{-}/(•\text{OH}/{\text{SO}}_{4}^{•-}/•\text{OOH}/{\text{O}}_{2}/{\text{O}}_{2}^{•},\ldots )\to \text{intermediates}+{\text{CO}}_{2}+{\text{H}}_{2}\text{O}+\text{inorganic ions}$$

BDD is incapable of absorbing organics (see Text S1 and Figure S1 for more details) and a nonactive anode, which can react with H_2_O to produce the physisorbed •OH (BDD(•OH)) (Eq. (2)), rending the organics degradation (Eq. (3)). The SO_4_^•−^ (Eq. (4)) also contributes to the degradation of organics.


(2) $$\text{BDD}+{\text{H}}_{2}\text{O}®\text{M}(•\text{OH})+{\text{H}}_{+}+{\text{e}}_{-}$$

(3) $$\text{BDD}(•\text{OH})+\text{R}®\text{BDD}+{\text{CO}}_{2}+{\text{H}}^{+}+{\text{e}}^{-}$$

(4) $${\text{SO}}_{4}^{2}®{\text{SO}}_{4}^{•-}+{\text{e}}^{-}$$

Herein, this work reported the degradation of CT (in Padan 95SP) on BDD electrode. The BDD electrode was selected in this study because it performs very good electrochemical capability (high stability, resistance to corrosion, high overpotential for oxygen evolution) [9–[10]^11^]. Padan 95SP was selected for the degradation in EO process because of its widespread use in pest control in Vietnam.

The kinetic degradation of CT can be expressed as:


(5) $$-\text{ln }\frac{{\left[\text{CT}\right]}_{0}}{{\left[\text{CT}\right]}_{t}}=k_{CT}t=(k_{•OH,\text{CT}}{\left[•\text{OH}\right]}_{ss}+k_{{SO}_{4}^{•-},\text{CT}}{\left[{\text{SO}}_{4}^{•-}\right]}_{ss}+k_{ROS}+e^{-})t,$$

where *k_CT_* represents the first-order rate constant of CT in the EO process, min^−1^; *k*_• _*_OH, CT_* and $k_{{SO}_{4}^{•-},\text{CT}}$ the second-order rate constant of CT toward •OH and SO_4_^•−^, respectively, (M^−1^ s^−1^); *k_ROS_* and *e^−^* represent the first-order rate constant of CT by ROS and DET, (min^−1^); [•OH]*_ss_* and ${\left[{\text{SO}}_{4}^{•-}\right]}_{ss}$ are the steady-state concentrations of •OH and SO_4_^•−^, respectively (M).

The purpose of this study was to: (1) investigate the direct and indirect oxidation of CT in EO process; (2) study the effect of water matrices on CT removal efficiency; (3) insight into the contribution of radicals to the degradation of CT.

## 2. Materials and methods

### 2.1. Chemicals

Commercial pesticide Padan 95SP (95% CT) was purchased in Vietnam. Ellman’s reagent (DTNB, 5,5′-Dithiobis (2-nitrobenzoic acid)) was supplied by Sigma-Aldrich for estimating the concentration of CT in Padan 95SP at interval times of electrolysis. Other chemicals (i.e. glycerol, benzoic acid (BA, 99%), nitrobenzene (NB, 99%), MeOH (99%), TBA (99%), boric acid, sodium sulfate, etc.) were also supplied by Sigma-Aldrich and Merck. The stuck solutions were prepared using deionized ultrapure water (Seralpur Pro 90 C).

### 2.1. BDD characterization

The BDD electrode was characterized using scanning electron microscopy (SEM, JSM-IT200, Japan), equipped with energy-dispersive X-ray spectroscopy (EDX) to analysis the elemental composition. X-ray diffraction (XRD, D8 ADVANCE ECO, Germany) with Cu Kα radiation (0.154 nm) was also used to determine the crystal structure of BDD.

### 2.2. Electrolysis

The electrolysis was carried out at ambient temperature (22 °C) in undivided cell of 400 mL. A BDD was used as the working electrode, supplied from Neocoat (Switzerland). The exposed surface area of BDD was 3.8 cm^2^, the diamond layer was about 2.5–3 μm. In all cases, a Platinum foil with the surface area of one side of 2 cm^2^ and Ag/AgCl (saturated KCl) were used as counter and reference electrodes, respectively. To stir 250 mL electrolyte solution continuously during the process, a magnetic bar was implied. Before starting experiment the working and counter electrodes were washed in ultrasonic bath for 10 min to remove contaminants, and then washed again with ultrapure water. The pH values of solution was controlled by 1 M H_2_SO_4_ or 1 M NaOH using a pH meter. Each experiment was duplicated for verification.

Electrolysis experiments were conducted under galvanostatic control at the applied current density ranged from 10 to 40 mA cm^−2^ using IviumStat (5 A current compliance/10 V power supply). Linear sweep voltammetry (LSV) test was performed in the potential range of 0–2 V at scan rate of 50 and 100 mV s^−1^ in 0.05 M Na_2_SO_4_. For CVs tests, the BDD electrode was characterized electrochemically in 0.05 M Na_2_SO_4_ solution in the absence and in the presence of Padan 95 SP. The cyclic voltammetry (CV) curves for the BDD electrode were recorded between −2.0 V to 2.0 V (*E^0^* vs. SCE) at different scan rates, sample interval was 0.01 V. The LSVs and CVs tests were conducted using Metrohm Autolab installed by Nova 2.1.3 software for electrochemical interface. Electrochemical impedance spectroscopy (EIS) was determine using Metrohm Autolab to investigate the conductivity of BDD in 0.2 M H_2_SO_4_ under the frequency from 1 × 10^5^ to 1 × 10^−2^ Hz at open circuit potential.

### 2.3. Analysis methods

The solution samples were withdrawn at the time intervals and immediately measured using DTNB procedure, which can be clearly described elsewhere [12,13]. This technique was based on the generated yellow anions (3-carboxy-4-nitrophenylthiolate anion), which were then determined at the maximum wavelength of 412 nm in a UV-Vis spectrophotometer (V730, Japan). The spectrum for CT determination can be seen in Text S2 and Figure S2.

The degradation efficiency of CT is calculated according to Eq. (6):


(6) $$m=\frac{1-C}{C_{0}}\times 100\%,$$

where *C* is the remaining content of CT at a given electrolytic time and *C_0_* is the initial concentration.

The theoretical mineralization of CT is proposed in Eq. (7):


(7) $${\text{C}}_{7}{\text{H}}_{15}{\text{N}}_{3}{\text{O}}_{2}{\text{S}}_{2}+20\;{\text{H}}_{2}\text{O}®2\;{\text{SO}}_{4}^{2-}+3\;{\text{NH}}_{4}^{-}+7\;{\text{CO}}_{2}+43{\text{H}}^{+}+42\;{\text{e}}^{-}.$$

The concentrations of BA and NB were measured by HPLC aligent 1200 (Germany) with C18 column (250 mm × 4.6 mm, 5 μm) coupled with a UV detector. The detection of BA and NB was performed at 227 and 270 nm, respectively, at a flow rate of 1 mL min^−1^. The mobile phase of methanol/water (65:35) (v/v) contained 1 % phosphoric acid. The column temperature was 30 ºC. Additionally, the concentration of BA and NB can be also calculated using UV-Vis (V730, Japan), because the formed byproducts and the presence of SO_4_^2−^ did not cause any interferes for the detection of BA and NB at 224 nm and 270 nm, respectively (The HPLC/UV spectrum of BA/NB and the degradation of BA/NB during the EO process can be seen in Figure S3).

## 3. Result and discussion

### 3.1. BDD characterization and electrochemical properties

**Figure 1:**
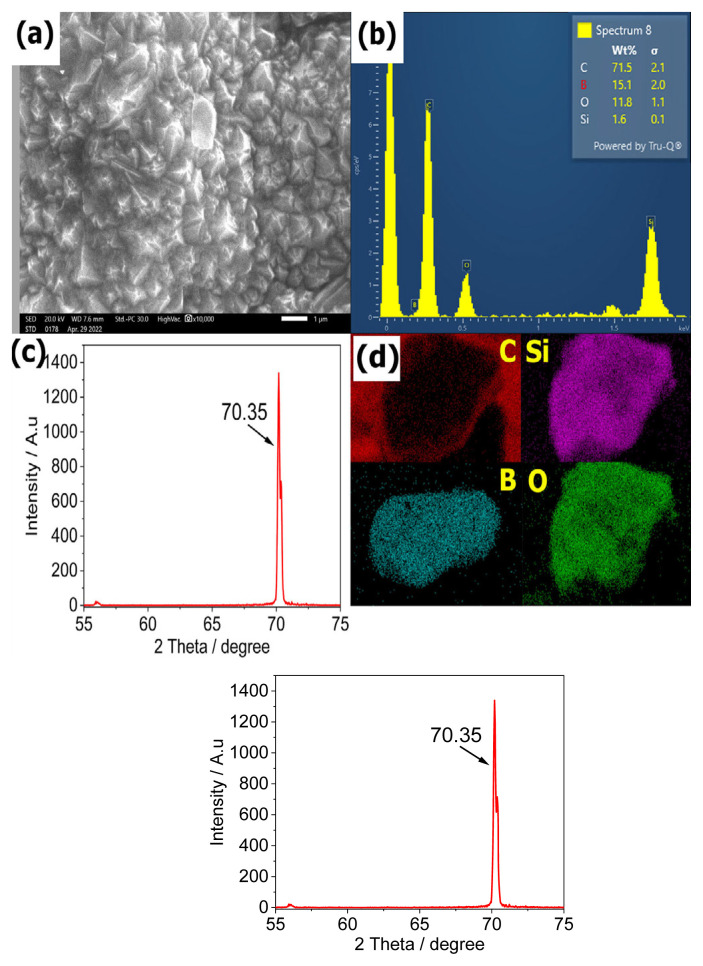
Elemental analysis for BDD electrode. a) SEM image of BDD; b) EDX for detecting elements; c) XRD patterns of the BDD electrode; d) Detected elements on BDD.

Due to the lack of information about this commercial BDD electrode, BDD was again characterized using SEM, XRD and EIS. As can be seen in Figure 1a, the BDD layer consists of the grains with the medium size of 200 nm. The orient of the grains was randomly grown on the substrate Si. According to EDX spectrum, some elements were detected, including C (71.5%), B (15.1%), O (11.8%), and Si (11.6%) (Figure 1b). Moreover, the phase of BDD can be proved by the diffraction peak at 2θ = 70.35°, which can be coincided with crystal planes of the hexoctahedral phase of diamond (diamond cubic) (Figure 1c). The roman spectrum and XPS of BDD can be clearly seen in our previous publication [10].

**Figure 2:**
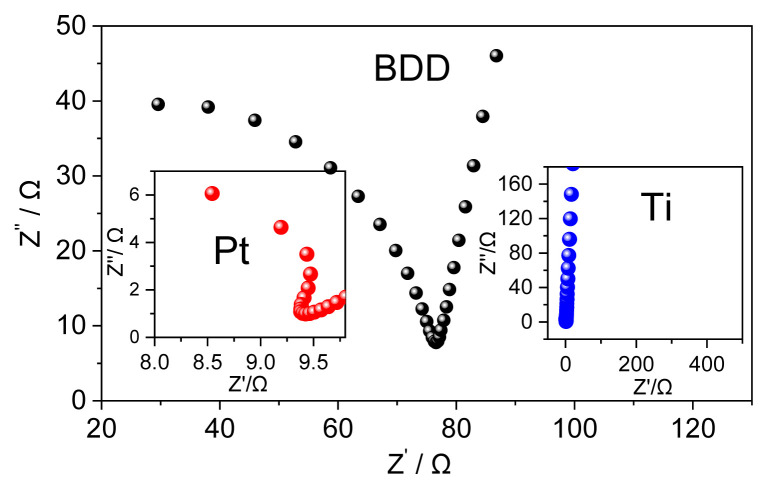
EIS spectrum of BDD. Inert: EIS spectrum of Pt and Ti.

The impedance spectrum of BDD and its comparison with Ti and Pt are shown in Figure 2. The arc diameter of BDD was much smaller than Ti and higher than Pt electrodes, indicating that charge transfer resistance of those electrodes followed the order: Ti (35 kΩ) > BDD (92.6 Ω) > Pt (13 Ω) (use the electrochemical circle fit command in NOVA 2.1.3 software to calculate the resistance). The above result indicates that the BDD can be considered a good conductive electrode thanks to the BDD layer on Si substrate.

### 3.2. Cyclic voltammetry curve in absence and presence of Padan 95 SP

**Figure 3:**
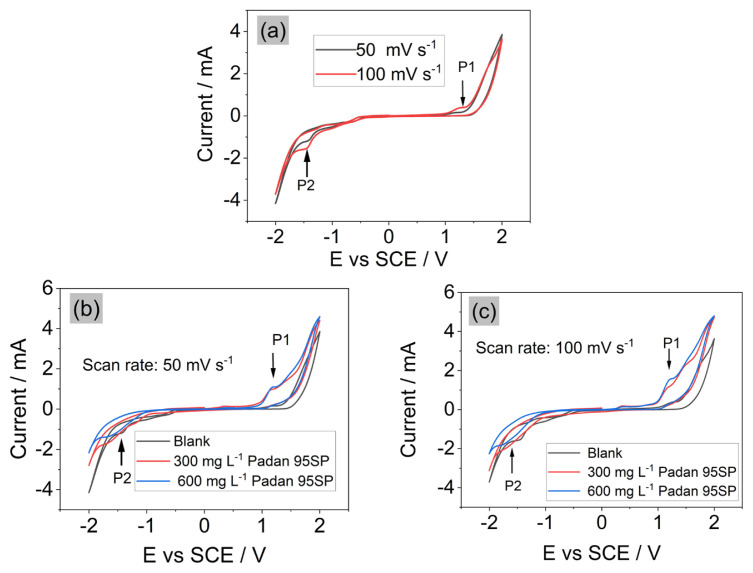
CVs of BDD in the presence of CT and Na_2_SO_4_. **a)** CVs of BDD in 300 mg L^−1^ CT at different scan rates. **b, c)** The comparison of CVs between blank solution and CT solution. 0.05 M Na_2_SO_4_ was used as supporting electrolyte.

The electrochemical character of BDD electrode with SO_4_^2−^ anion is an important factor to initially study the CT degradation mechanism in Na_2_SO_4_. The CV curves of BDD electrode under different conditions are depicted in Figure 3. Similar to previous study [14], we also found the oxidation peak **P1** occurring at the potential between 1 V and 1.3 V (Figure 3a), suggesting that the direct oxidation of CT can occur due to the electron transfer on the BDD surface. Generally, the oxidation peak potential must be less than the oxygen evolution potential (Eq. (8)), as also confirmed by M. Panizza and G. Cerisola [8]:


(8) $${\text{H}}_{2}\text{O}®4{\text{H}}^{+}+4{\text{e}}^{-}+{\text{O}}_{2}\;\;\;{\text{E}}^{0}=1.23\;\text{V }({\text{E}}^{0}\;\text{vs NHE})$$

When increasing the scan rate to 100 mV s^−1^, the direct oxidation of CT was enhanced, as a result of the promotion of electron exchange at BDD surface. This is because the high scan rates provides high current density for shorter time frame, thereby increasing the oxidation current peak [15]. Therefore, the degradation of CT underwent both mechanisms: (1) direct oxidation via electron transfer at the surface of BDD; (2) the indirect oxidation by reactive radicals. The contribution of direct oxidation and indirect oxidation (via •OH, SO_4_^•−^, etc.) were discussed in subsections 3.4 and 3.5.

In the negative potential, the reduction peak **P2** at −1.2 V is probably associated to the evolution of H_2_ from H_2_O and/or the formation of persulfate (Eqs. (9) and (10)). When increasing the scan rate to 100 mV s^−1^, the intensity of the reduction peak increased.


(9) $${\text{SO}}_{4}^{•-}+{\text{SO}}_{4}^{•-}®{\text{S}}_{2}{\text{O}}_{8}^{2-}$$

(10) $${\text{SO}}_{4}^{•-}+{\text{SO}}_{4}^{2-}®{\text{S}}_{2}{\text{O}}_{8}^{2-}+{\text{e}}^{-}$$

The further investigation of the scan rates at different concentrations of CT were illustrated in Figures 3b and 3c and there are no significant differences between two ranges of concentration, indicating that the degradation through direct oxidation is unchanged at high CT concentration.

In addition, the effect of current density on O_2_ evolution and CT degradation can be found in detail in Texts S3 and S4, Figures S4–S6.

### 3.3. Effect of different water matrices

**Figure 4:**
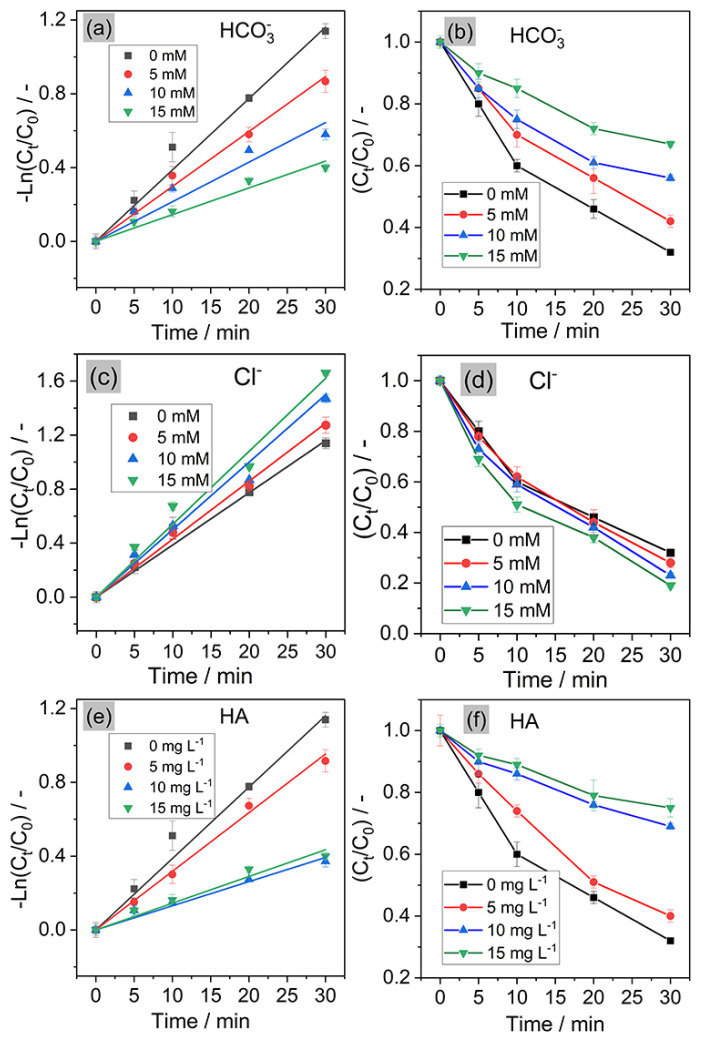
Effect of HCO_3_^−^, Cl^−^, and HA on CT degradation in electrochemical process. (a, c, e) The first-order kinetics of CT in the presence of HCO_3_^−^, Cl^−^, and HA, respectively. (b, d, f) Relative degradation of CT in the presence of HCO_3_^−^, Cl^−^, and HA, respectively. Experimental conditions: pH = 3, current density *j* = 40 mA cm^−2^, [CT] = 40 μM, [Na_2_SO_4_] = 0.05 M.

Some anions (i.e. HCO_3_^−^/CO_3_^2−^, Cl^−^, Fe^2+^, NO_3_^−^, etc.) and humic acid (HA) can be found in natural water. In this study, HCO_3_^−^ and Cl^−^ were selected because they are common anions in water. HA was chosen because it represents the organic matter in natural water. Their presence affects the degradation efficiency of organics during the process. The formation of radicals and their transformation in the presence of water matrices can be listed in Table S2. Therefore, the effects of HCO_3_^−^, Cl^−^ and HA on CT removal were investigated, as shown in Figure 4.

*Effect of HCO_3_^−^: k_CT_* (the first-order rate constant for CT) decreased from 0.039 min^−1^ to 0.014 min^−1^ (decreased by 74%) when increasing HCO_3_^−^ concentration from 0 to 15 mM (Figure 4a). This result could be attributed to the scavenging effect of bicarbonate toward •OH and SO_4_^•−^ (Reactions (82) and (83), Table S2). This scavenging effect leads to the formation of CO_3_^•−^ with weaker oxidation capability (their reaction rate constant toward organics is in the range of 10^−6^–10^−7^ M^−1^ s^−1^, Table S2), thereby reducing the removal efficiency of process. As a result, CT removal decreased from 68% to 32% after 30 min when increasing HCO_3_^−^ concentration from 0 to 15 mM (Figure 4b).

*Effect of Cl^−^: k_CT_* increased from 0.039 min^−1^ to 0.054 min^−1^ (increased by 1.4 folds) when increasing Cl^−^ concentration from 0 to 15 mM (Figure 4c). The removal efficiency increased from 68% to 81% after 30 min as increasing Cl^−^ concentration to 15 mM (Figure 4d). The presence of chloride ion can enhance the CT removal due to some aspects: (1) Cl^−^ could be oxidized by electrolysis to produce active chlorine and/or chloride radicals (ClO^−^/Cl•) [16,17]. The oxidation capability of Cl• toward organics is comparable to •OH (in the range of 10^9^–10^10^ M^−1^ s^−1^. Table S2), thereby contributing to the degradation of CT; (2) Cl^−^ reacts with •OH and SO_4_^•−^ to produce reactive chlorine species (RCS: Cl•, Cl_2_^•−^, ClOH^•−^, etc) (Reactions 35–80, Table S2) which could also enhance the removal efficiency of CT in the electrolysis process. Despite the fact that the scavenging effect of Cl^−^ lead to decreased concentration of SO_4_^•−^, the significant formation of RCS at high concentration of Cl^−^ might compensate the fade of SO_4_^•−^, leading to the improvement of process. The same results can be observed in other AOPs [18–21], where the kinetic formation of radicals can be similarly observed.

*Effect of HA*: HA acts as scavenger of oxidizing species, leading to a reduction in the degradation efficiency. As can be seen in Figure 4e, increasing the concentration of HA to 15 mg L^−1^ reduced *k_CT_* from 0.039 min^−1^ to 0.014 min^−1^ (decreased by 64%), and the removal efficiency decreased from 68% to 23% after 30 min. This result can be explained by several mechanisms [16]: (1) HA competed with CT for the adsorption at the Pt cathode and BDD anode, thus, less CT was degraded at the surface of electrodes; (2) HA competed with sulfate ion on the BDD anode, leading to a reduction in the formation of SO_4_^•−^ (Eq. (4)); (3) HA caused the scavenging effect toward •OH and SO_4_^•−^ (Reactions (101) and (102), Table S2).

### 3.4. Determination of reactive species

**Figure 5:**
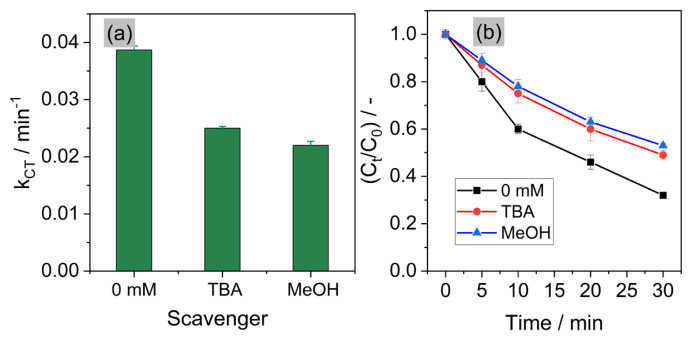
a) The first-order rate constant (*k_CT_*) of CT degradation in the presence of TBA and MeOH; b) Relative degradation of CT in the presence of TBA and MeOH. Experimental conditions: pH = 3, current density *j* = 40 mA cm^−2^, [CT] = 40 μM, [TBA] = [MeOH] = 100 mM, [Na_2_SO_4_] = 0.05 M.

Generally, •OH and SO_4_^•−^ were found to be major radicals in EO process [22]. To determine the role of these radicals to CT degradation, TBA used as a scavenger for •OH (*k*_•_*_OH,TRA_* = (3.8−7.6)×10^8^ M^−1^ s^−1^) [16]. The reaction between SO_4_^•−^ and TBA can be ignored ( $k_{{SO}_{4}^{•-},TBA}=(4-9.1)\times 10^{5}\;{\text{M}}^{-1}\;{\text{s}}^{-1}$) [16]. MeOH was used as probe for •OH (9.7 × 40^8^ M^−1^ s^−1^) and SO_4_^•−^ (1.0 ×10^7^ M^−1^ s^−1^). As seen in Figure 5a, *k_CT_* decreased by 35% and 43% when adding 100 mM TBA and MeOH, respectively. CT removal efficiency decreased from 68% to 37% and to 29% after 30 min at 100 mM TBA and MeOH, respectively (Figure 5b). The significant decrease in *k_CT_*, and *k_CT_* (in TBA) > *k_CT_* (in MeOH) suggested that •OH and SO_4_^•−^ were the major radicals contributing to CT degradation. In addition, the other ROS ( ${\text{O}}_{2}^{•-}$, O^•−^, ${\text{HO}}_{2}^{•}$, etc.) (Reactions (4–25), Table S2) could also form in the electrolysis process as the reaction chains in the solution. However, their role on CT degradation was assumed to be negligible due to their low concentration as suggested by [16].

### 3.5. The relative contribution of reactive species to CT degradation

**Figure 6:**
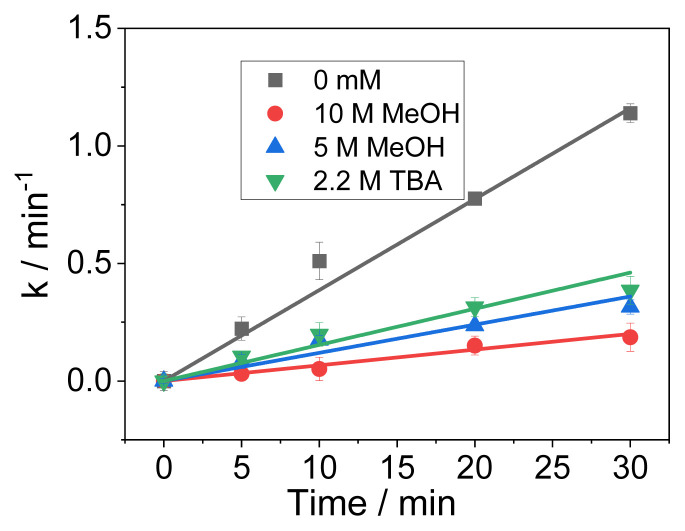
Reaction kinetics of CT in the presence of probes. Experimental conditions: pH = 3, current density *j* = 40 mA cm^−2^, [CT] = 40 μM, [Na_2_SO_4_] = 0.05 M.

In EO process, the oxidation of CT can be taken placed by several oxidizing factors as displayed in Eq. (5): •OH, SO_4_^•−^, other ROS, and DET. To calculate the degradation of CT by DET, MeOH was used to eliminate the effect of ROS. Assuming that adding 10 M MeOH could scavenge all ROS (including •OH, SO_4_^•−^) completely, then *k_DET_* was calculated to be 0.006 min^−1^, accounted for 15% of CT degradation (Figure 6). The change in *k_CT_* with addition of scavengers can be seen in Figure 6. •OH and SO_4_^•−^ could be generated by electrolysis alone at the surface of anode (Eqs. (2) and (4)) and/or from the reactions chains in solution (Reactions 1–29, Table S2). The relative contribution of •OH and SO_4_^•−^ was calculated according to Eqs. (11) and (12) [22]. As a result, •OH and SO_4_^•−^ accounted for 61.5% and 12.5%, respectively.


(11) $$\eta_{•OH}=\frac{k_{CT}-k_{TBA}}{k_{CT}}\times 100\%$$

(12) $$\eta_{SO_{4}•-}=\frac{k_{MeOH}-k_{TBA}}{k_{CT}}\times 100\%$$

where *k_CT_* represents the degradation rate constant of CT without addition of scavenger, min^−1^; *k_TBA_* represents the degradation rate constant of CT with addition of TBA (2.2 mM), min^−1^; *k_MeOH_* is the degradation rate constant with addition of MeOH (5 mM), min^−1^.

Furthermore, to estimate the steady-state concentration of •OH and SO_4_^•−^, NB and BA were used because NB can react with •OH (*k*_•_*_OH_*_, NB_ = 3.9 × 10^9^ M^−1^ s^−1^) and its reactivity toward SO_4_^•−^ was negligible ( $k_{{SO}_{4}^{•-},NB}<10^{6}\;{\text{M}}^{-1}\;{\text{s}}^{-1}$) [23]. BA can react with both •OH and SO_4_^•−^ (*k*_•_*_OH_*_, _*_BA_* = 5.9 × 10^9^ M^−1^ s^−1^ [24], ( $k_{{\text{SO}}_{4}^{•-},\text{BA}}=1.2\times 10^{9}{\text{M}}^{-1}\;{\text{s}}^{-1}$) [25]. Therefore, the degradation of NB and BA in EO process can be expressed as follows [26]:


(13) $$k_{NB}=k_{e}+k_{•OH,NB}{\left[•\text{OH}\right]}_{ss}$$

(14) $$k_{BA}=k_{e}+k_{•OH,BA}{\left[•\text{OH}\right]}_{ss}+k_{{SO}_{4}^{•-},BA}{\left[{\text{SO}}_{4}^{•-}\right]}_{ss}.$$

Assuming that the degradation of NB and BA by DET is negligible, the above reactions can be rewritten:


(15) $$k_{NB}=k_{•OH,NB}{\left[•\text{OH}\right]}_{ss}$$

(16) $$k_{BA}=k_{•OH,BA}{\left[•\text{OH}\right]}_{ss}+k_{{SO}_{4}^{•-},BA}{\left[{\text{SO}}_{4}^{•-}\right]}_{ss},$$

where *k_NB_*, *k_BA_* (min^−1^) represent the degradation of NB, BA in EO process (Experimental conditions were carried out similar to CT degradation: pH = 3, current density *j* = 40 mA cm^−2^, [NB] = [BA] = 40 μM, [Na_2_SO_4_] = 50 mM, see Figure S3). By solving the reactions (15) and (16), [•OH]*_ss_* and ${\left[{\text{SO}}_{4}^{•-}\right]}_{ss}$ were estimated to be 3.2 × 10^−13^ M and 5.8 × 10^−14^ M, respectively.

The overview of the contribution of oxidants to CT degradation was listed in Table.

### 3.6. Transformation and interaction mechanism of radicals

It is known that the degradation of organics was performed mainly by •OH radicals, so the mechanism of generating hydroxyl radicals on the anode surface is written as follows:


(17) $$\text{BDD}+{\text{H}}_{2}\text{O}®\text{BDD}(•\text{OH})+{\text{H}}^{+}+{\text{e}}^{-}$$

(18) $$\text{BDD}(•\text{OH)}+\text{CT}®\text{CT}•+\text{BDD}+{\text{H}}^{+}+\text{e}$$

(19) $$\text{BDD}(•\text{OH)}+\text{CT}•®\text{BDD}+\text{intermediates}+{\text{CO}}_{2}+{\text{H}}_{2}\text{O}\text{.}$$

After losing electrons at the surface of BDD the H_2_O molecules generate •OH radical (Eq. (17)). Hydroxyl radical is a strong oxidant, which can attack CT rapidly to generate radical CT•. As a low stable radical, CT• reacts with hydroxyl radical to further generate new intermediates and the end products (i.e. CO_2_ and H_2_O). Besides, the formation of SO_4_^•−^ can take place through electron-transfer on the surface of BDD and/or from •OH. SO_4_^•−^ then attacks CT to reproduce SO_4_^2−^ and cationic radical CT^+•^, as described in Eqs. (20) and (21). SO_4_^•−^ is considered as very active oxidant (*E^0^* = 2.6 V), can further destroy this organic radical to the end products, or generate smaller molecules.


(20) $${\text{HSO}}_{4}^{-}+•\text{OH}®{\text{SO}}_{4}^{•-}+{\text{H}}_{2}\text{O}$$

(21) $${\text{SO}}_{4}^{•-}+\text{CT}®{\text{CT}}^{+•}+{\text{H}}^{+}+{\text{SO}}_{4}^{2-}$$

(22) $${\text{SO}}_{4}^{•-}+{\text{CT}}^{+□}®{\text{SO}}_{4}^{2-}+{\text{CO}}_{2}+{\text{H}}_{2}\text{O}$$

Additionally, some mechanisms involving with this radical may occur, as described in the reactions below:


(23) $${\text{SO}}_{4}^{•-}+{\text{SO}}_{4}^{•-}®{\text{S}}_{2}{\text{O}}_{8}^{2-}$$

(24) $${\text{SO}}_{4}^{2-}®{\text{SO}}_{4}^{•-}+{\text{e}}^{-}$$

(25) $${\text{HSO}}_{4}^{-}®{\text{SO}}_{4}^{•-}+{\text{H}}^{+}+{\text{e}}^{-}$$

(26) $${\text{SO}}_{4}^{•-}+{\text{SO}}_{4}^{2-}®{\text{S}}_{2}{\text{O}}_{8}^{2-}+{\text{e}}^{-}$$

(27) $${\text{SO}}_{4}^{•-}+{\text{SO}}_{4}^{2-}®{\text{S}}_{2}{\text{O}}_{8}^{2-}+{\text{H}}^{+}+{\text{e}}^{-}.$$

In the presence of •OH, protonized ${\text{HSO}}_{4}^{-}$ could be oxidized to form SO_4_^•−^ (Eq. (20)), which can recombine (Eq. (23)) [27,28] or further react with sulfate and/or hydrosulfate ions to form persulfate S_2_O_8_^2−^ (Eqs. (26) and (27)) [29,30], as confirmed by the work of F. Zhang et al. [14] using ESR spectrum. Furthermore, the evolution of O_2_ takes place via a recombination of •OH as in Eq. (28) or from the hydrolysis of persulfate (Eq. (29)):


(28) $$•\text{OH}+•\text{OH}®{\text{O}}_{2}+2{\text{H}}^{+}+2{\text{e}}^{-}$$

(29) $${\text{S}}_{2}{\text{O}}_{8}^{2□}+{\text{H}}_{2}\text{O}®{\text{HSO}}_{4}^{•-}+1/2{\text{O}}_{2}.$$

**Figure 7:**
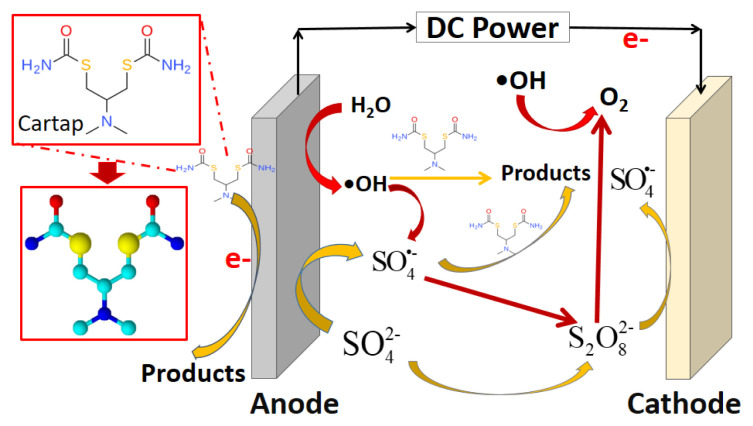
The electrochemical oxidation of CT and the transformation of radicals.

It is worth nothing that the electrochemical degradation of organics is a complex of various mechanisms, in which the oxidizing radicals could be produced by different ways, involving in the degradation of organics as well as their none-used disappearance. Briefly, we can see the whole process of oxidizing CT in Figure 7. Additionally, the degradation pathway of CT in EO process can be found in our previous study [17].

## 4. Conclusion

The kinetic electrochemical degradation of CT (in Padan 95 SP) was investigated. The degradation of CT underwent two mechanisms: direct oxidation by electron transfer and indirect oxidation by reactive generated species. The removal efficiency of CT depended on the presence of water matrices: 15 mM Cl^−^ improved *k_CT_* by 38%, while *k_CT_* was reduced by 61.5% and 64% when adding 15 mM HCO_3_^−^ and 15 mg L^−1^ HA, respectively. CT removal efficiency was reduced in the presence of MeOH and TBA, indicating that reactive species play an important role in CT degradation. The contribution of reactive oxidants was established: DFT accounted for 15%; •OH accounted for 61.5%; SO_4_^•−^ accounted for 12.8%; ROS accounted for 8.5%. As a small part of study, the effect of applied current on CT degradation was investigated, indicating the O_2_ evolution at higher current inhibited the CT removal. In addition, the possible transformation pathways of reactive species was suggested.

## Supporting information

### Text S1: adsorptive possibility of BDD toward organic compounds

To check the change in concentration of CT and organic compounds in the presence of BDD without applied potential, different types of organic compounds were used. Those chosen compounds are dye compounds, which can be adsorbed high by some adsorbent (i.e. active carbon). However, there was no any reduction in concentration of CT and dye compounds (Figure S1), indicating that BDD is a nonadsorptive electrode.

### Text S2: spectrum for Cartap detection

The absorbance spectrum of standard CT (in Padan 95SP) in the ranges of interested concentration were recorded in Figure S2 to establish the calibration curve for assessing the degradation of CT. It is also noted that the absorbance spectrum from CT decomposition during electrochemical process did not differ with the presence of other species (i.e. intermediates, electrolytes and pH). Condition for this measurement: 0.2 mL each trail was mixed well with 0.8 mL DTNB solution (1 g L^−1^ DTNB in methanol) and then with 4 mL buffer solution, as can be seen in detail elsewhere for preparing the sample for UV-Vis measurement [1,2].

### Text S3: linear polarization curve

As a side reaction in electrochemical degradation of CT, the oxygen formation at BDD was assessed via linear sweep voltammetry (LSV). Thus, the linear polarization curves of BDD electrode was tested at two different scan rates of 50 and 100 mV s^−1^ in 0.05 M Na_2_SO_4_ electrolyte solution, as shown in Figure S4.

It is argued that the oxygen evolution reaction (OER) occurs since the current passing the electrode suddenly increases. Figure S4 shows that the higher OER (1.6 V) can be achieved at slower scan rate (50 mV s^−1^), indicating the dependence of OER on scan rate. Additionally, the oxygen evolution overvoltage of both cases is pretty lower than that we expected from BDD. It should be kept in mind that the potential for oxygen evolution at anode is just a relative value and it might also differ under the experimental conditions. For example, Costa et al. [3] have pointed out that OER even occurred at the potential less than 2 V vs. SCE at 0.01 M H_2_SO_4_. They also showed a strong dependence of OER on the organic target, which contributes to a decrease in the potential of oxygen evolution when increasing its concentration. Similarly, O. Davila et al. figured out that the onset potentials for OER in electrolysis of BDD depends on the mixtures with dibenzothiophene (67 mg L^−1^ at scan rate: 10 mV s^−1^) [4]. Some authors argued that the oxygen evolution rate decreased when adding organic compounds due to their competition with •OH [5,6]. In our condition, it is not an exceptional when we observed the low EOR. This might be due to the low concentration of supporting electrode (here: 0.05 M Na_2_SO_4_), dominating the oxygen evolution instead of oxidizing sulfate ions to other species. However, the anodic current is low within the potential interval from 1.50 to 2 V, indicating that not much oxygen was generated. The oxygen evolution at some BDD anodes are depicted in Table S1 below:

Figure S1. Concentration of organic compounds before and after immersed BDD electrode: CT (Cartap), MB (methylene blue), Ind (indigo carmine), RNO (P-nitrosodimethylaniline). The initial concentration of organic compounds was 40 μM, *V_solution_* = 100 mL.

Figure S2. The intensity of absorbance spectra for calculating CT concentration in water. Insert: Calibration plot for calculating CT concentration based on data of Figure 2.

Figure S3 The kinetic degradation of NB and BA (by HPLC detection (A) and by UV-Vis detection (B)) to determine the concentration of •OH and SO_4_•-. Experimental conditions: [NB] = [BA] = 40 μM, [Na_2_SO_4_] = 0.05 M, pH = 3, *V* = 250 mL, current density *j* = 40 mA cm^−2^.

### Text S4: effect of applied current density

The effect of the applied current density (*j*) on the CT degradation in the undivided cell is displayed in Figure S5.

As shown in Figure S5, the CT degradation rate increased at higher applied current density. More clearly, this result shows about 70% CT removed after 30 min at *j* = 40 mA cm^−2^, meanwhile only 57% at *j* = 10 mA cm^−2^. The result shows that this process fitted well the pseudo-first order kinetic, which is described by the Eq. (S1):


(S1) $$\text{ln}(\frac{C_{t}}{C_{0}})=□k_{CT}t,$$

where *C_t_* and *C_0_* are the concentration of CT at the interval time (*t*) and the beginning time, respectively. *k_CT_* is the apparent rate constant of CT. We perform the degradation kinetic of CT in Figure S5b to determine the change in the *k_CT_* versus current density. Obviously, *k_CT_* increased from 0.022 min^−1^ to 0.039 min^−1^ (increased by 1.7-folds) when increasing the current density by 4 folds, suggesting that a production of •OH radicals becomes inefficiently at higher current due to the competing reactions, here is oxygen evolution [10] (see Eq. (S2)). The fact can be demonstrated by mineralization current efficiency (MCE) in our previous paper [11]. This bubble gas can also deactivate surface of anode, thereby reducing the removal efficiency.


(S2) BDD(•OH)□BDD+12 O2+H++e-

Additionally, based on the calculation results for *k_CT_* from Figures S5 and S6, and the suggestion of reference [12], we propose the relationship between the degradation rate constant *k_CT_* and the applied current density *j* as the quadratic function, as shown in Figure S6. This function fits well our experimental results ranging from 10 mA cm^−2^ to 40 mA cm^−2^ with *R-square* of 0.978. Thus, we propose this relationship in Eq. (S3).


(S3) $$k_{CT}=2□10^{-5}\;j^{2}+5.8□10^{-4}j+0.019$$

Substitute the value *k_CT_* from Eq. (1) into Eq. (S3), we obtain the kinetic reaction:


(S4) $$□\text{ln}(\frac{C_{t}}{C_{0}})=(2□10^{-5}\;j^{2}+5.8□10^{-4}j+0.019)t,$$

where *C_t_* and *C_0_* (μM) are concentration of CT at time intervals *t* (min) and at the beginning, respectively. *j* is the current density (mA cm^−1^).

Figure S4. LSV of BDD in 0.05 M Na_2_SO_4_ at two scan rates. pH = 6.5 and at room temperature.

Figure S5 **a)** Effect of *j* on CT degradation as a function of time (*j* from 10 to 40 mA cm^−2^). **b)** Effect of *j* on the apparent rate constant. Initial concentration of Padan 95SP (95% CT): 40 μM, supporting electrolyte: 0.05 M Na_2_SO_4_, pH = 3, BDD: working electrode.

Figure S6. The plot of degradation rate constant versus current density.

**Table S1.** Potential for oxygen evolution on electrodes.

Anode	E ^0^	Conditions	References
Ti/BDD	2.7 V vs. SHE	0.5 M H _2_SO _4_	-
Mesh BDD	1.95 V vs. SCE	0.03 M Na _2_SO _4_, scan rate: 20 mV s ^−1^	[ 7]
BDD	1.5 V vs. SCE	1 M H _2_SO _4_, scan rate: 20 mV s ^−1^	[ 8]
BDD	0.9 V vs. SCE	0.25 M NaOH + 0.5 M Na _2_SO _4_, scan rate: 100 mV s ^−1^	[ 9]
BDD	2 V vs. SCE	0.5 M H _2_SO _4_, scan rate: 100 mV s ^−1^	[ 9]

**Table S2.** The kinetic model in electrochemical system.

No.	Reaction	*k* (M ^−1^s ^−1^)/s ^−1^	Reference
**HO** **_X_** **^•^** ** and SO** **_X_** **^•−^** ** reactions**			
1	SO _4_ ^•−^ + SO _4_ ^•−^ → S _2_O ^82^-	4.0 × 10 ^8^ M ^−1^s ^−1^	[ 13]
2	SO _4_ ^•−^ + OH ^−^ → ^•^OH + SO _4_ ^2−^	6.5 × 10 ^7^ M ^−1^s ^−1^	[ 13]
3	^•^OH + ^•^OH → H _2_O _2_	6.1 × 10 ^9^ M ^−1^s ^−1^	[ 14]
4	^•^OH + OH ^−^ → O ^•−^+ H _2_O	1.2 × 10 ^10^ M ^−1^s ^−1^	[ 15]
5	^•^OH + SO _4_ ^•−^ → HSO _5_ ^−^	1.0 × 10 ^10^ M ^−1^s ^−1^	[ 13]
6	^•^OH + H _2_O _2_ → HO _2_ ^•^ + H _2_O	2.7 × 10 ^7^ M ^−1^s ^−1^	[ 13]
7	HO _2_ ^•^ + HO _2_ ^•^ → H _2_O _2_ + O _2_	8.3 × 10 ^5^ M ^−1^s ^−1^	[ 14]
8	HO _2_ ^•^ → O _2_ ^•−^ + H ^+^	7.0 × 10 ^5^ s ^−1^	[ 16]
9	H _2_O _2_ → HO _2_ ^−^ + H ^+^	1.3 × 10 ^−1^ s ^−1^	[ 14]
10	H _2_O _2_ + O _2_ ^•−^ → ^•^OH + OH ^−^ + O _2_	1.3 × 10 ^−1^ M ^−1^s ^−1^	[ 17]
11	H _2_O _2_ + O ^•−^ → O _2_ ^•−^ + H _2_O	4.0 × 10 ^8^ M ^−1^s ^−1^	[ 15]
12	HO _2_ ^−^ + H ^+^ → H _2_O _2_	5.0 × 10 ^10^ M ^−1^s ^−1^	[ 14]
13	H _2_O → H ^+^ + OH ^−^	1.0 × 10 ^−3^ M ^−1^s ^−1^	[ 14]
14	H ^+^ + OH ^−^ → H _2_O	1.0 × 10 ^11^ M ^−1^s ^−1^	[ 14]
15	O _2_ ^•−^ + H ^+^ → HO _2_ ^•^	5.0 × 10 ^10^ M ^−1^s ^−1^	[ 16]
16	HO _2_ ^•^ + O _2_ ^•−^ → HO _2_ ^−^ + O _2_	9.7 × 10 ^7^ M ^−1^s ^−1^	[ 16]
17	^•^OH + HO _2_ ^•^ → H _2_O + O _2_	7.1 × 10 ^9^ M ^−1^s ^−1^	[ 16]
18	^•^OH + O _2_ ^•−^ → OH ^−^ + O _2_	1.0 × 10 ^10^ M ^−1^s ^−1^	[ 16]
19	^•^OH + O ^•−^ → HO _2_ ^−^	1.0 × 10 ^10^ M ^−1^s ^−1^	[ 15]
20	^•^OH + HO _2_ ^−^ → H _2_O + O _2_ ^•−^	7.5 × 10 ^9^ M ^−1^s ^−1^	[ 13]
21	^•^OH + HSO _5_ ^−^ → SO _5_ ^•−^ + H _2_O	1.7 × 10 ^7^ M ^−1^s ^−1^	[ 13]
22	SO _4_ ^•−^ + HSO _5_ ^−^ → SO _5_ ^•−^ + HSO ^4^-	1.0 × 10 ^5^ M ^−1^s ^−1^	[ 18]
23	SO _4_ ^•−^ + S _2_O _8_ ^2−^ → SO _4_ ^2−^ + S _2_O ^8•^-	6.1 × 10 ^5^ M ^−1^s ^−1^	[ 18]
24	SO _4_ ^•−^ + H _2_O _2_ → HO _2_ ^•^ + HSO ^4^-	1.2 × 10 ^7^ M ^−1^s ^−1^	[ 13]
25	SO _4_ ^•−^ + H _2_O _2_ → HO _2_ ^•^ + H ^+^ + SO _4_ ^2−^	1.2 × 10 ^7^ M ^−1^s ^−1^	[ 19]
26	HSO _4_ ^−^ + ^•^OH → SO _4_ ^•−^ + H _2_O	6.9 × 10 ^5^ M ^−1^s ^−1^	[ 13]
27	2SO _5_ ^•−^ → 2SO _4_ ^•−^ + O _2_	1.0 × 10 ^8^ s ^−1^	[ 13]
28	2SO _5_ ^•−^ → S _2_O _8_ ^2−^ + O _2_	2.2 × 10 ^8^ s ^−1^	[ 20]
29	HSO _4_ ^−^ → SO _4_ ^2−^ + H ^+^	1.2 × 10 ^−2^ M ^−1^s ^−1^	[ 13]
**CT reactions**			
	**Primary radicals with CT**		
30	^•^OH + CT → pro	10 ^9^–10 ^10^ M ^−1^s ^−1^	Estimated
31	SO _4_ ^•−^ + CT → pro + SO _4_ ^2−^	10 ^9^–10 ^10^ M ^−1^s ^−1^	Estimated
	**Secondary radicals with CT**		
32	Cl ^•^ + CT → pro	10 ^9^–10 ^10^ M ^−1^s ^−1^	Estimated
33	Cl _2_ ^•−^ + CT → pro	-	-
34	CO _3_ ^•−^ + CT → pro	10 ^6^–10 ^7^ M ^−1^s ^−1^	Estimated
**Chloride reactions**			
35	SO _4_ ^•−^ + Cl ^−^ → SO _4_ ^2−^ + Cl ^•^	4.7 × 10 ^8^ M ^−1^s ^−1^	[ 18]
36	SO _4_ ^2−^ + Cl ^•^ → SO _4_ ^•−^ + Cl ^−^	2.5 × 10 ^8^ M ^−1^s ^−1^	[ 18]
37	Cl ^−^ + ^•^OH → ClOH ^•−^	4.3 × 10 ^9^ M ^−1^s ^−1^	[ 14]
38	ClOH ^•−^ + Cl ^−^ → Cl _2_ ^•−^ + OH ^−^	1.0 × 10 ^5^ M ^−1^s ^−1^	[ 14]
39	ClOH ^•−^ → Cl ^−^ + ^•^OH	6.1 × 10 ^9^ M ^−1^s ^−1^	[ 14]
40	ClOH ^•−^ + H ^+^ → Cl ^•^ + H _2_O	2.1 × 10 ^10^ M ^−1^s ^−1^	[ 14]
41	Cl ^−^ + Cl ^•^ → Cl _2_ ^•−^	6.5 × 10 ^9^ M ^−1^s ^−1^	[ 14]
42	Cl ^•^ + Cl ^•^ → Cl _2_	1.0 × 10 ^8^ M ^−1^s ^−1^	[ 14]
43	Cl ^•^ + H _2_O → HClOH	2.5 × 10 ^5^ M ^−1^s ^−1^	[ 14]
44	Cl ^•^ + H _2_O → ClOH ^•−^ + H ^+^	1.6 × 10 ^5^ M ^−1^s ^−1^	[ 14]
45	Cl _2_ ^•−^ + OH ^−^ → ClOH ^•−^ + Cl ^−^	4.5 × 10 ^7^ M ^−1^s ^−1^	[ 14]
46	Cl ^•^ + OH ^−^ → ClOH ^•−^	1.8 × 10 ^10^ M ^−1^s ^−1^	[ 14]
47	H ^+^ + Cl ^−^ → HCl	5.0 × 10 ^10^ M ^−1^s ^−1^	[ 14]
48	HCl → H ^+^ + Cl ^−^	8.6 × 10 ^16^ M ^−1^s ^−1^	[ 14]
49	Cl ^−^ + Cl _2_ → Cl _3_ ^−^	2.0 × 10 ^4^ M ^−1^s ^−1^	[ 14]
50	Cl _2_ ^•−^ → Cl ^•^ + Cl ^−^	1.1 × 10 ^5^ M ^−1^s ^−1^	[ 14]
51	Cl _2_ ^•−^ + Cl _2_ ^•−^ → Cl _2_ + 2Cl ^−^	8.3 × 10 ^8^ M ^−1^s ^−1^	[ 14]
52	Cl ^•^ + H _2_O _2_ → HCl + HO _2_ ^•^	4.0 × 10 ^9^ M ^−1^s ^−1^	[ 14]
53	Cl _2_ ^•−^ + H _2_O _2_ → 2Cl ^−^ + HO _2_ ^•^ + H ^+^	1.4 × 10 ^5^ M ^−1^s ^−1^	[ 16]
54	Cl _2_ ^•−^ + H _2_O → HClOH + Cl ^−^	1.3 × 10 ^3^ M ^−1^s ^−1^	[ 14]
55	Cl _2_ ^•−^ + ^•^OH → HOCl + Cl ^−^	1.0 × 10 ^9^ M ^−1^s ^−1^	[ 14]
56	Cl ^−^ + HOCl → Cl _2_OH ^−^	1.5 × 10 ^4^ M ^−1^s ^−1^	[ 16]
57	Cl _2_ ^•−^ + O _2_ ^•−^ → 2Cl ^−^ + O _2_	1.0 × 10 ^9^ M ^−1^s ^−1^	[ 14]
58	HOCl → H ^+^ + ClO ^−^	1.6 × 10 ^3^ s ^−1^	[ 14]
59	H ^+^ + ClO ^−^ → HOCl	5.0 × 10 ^10^ M ^−1^s ^−1^	[ 14]
60	HClOH → ClOH ^•−^ + H ^+^	1.0 × 10 ^8^ s ^−1^	[ 14]
61	HClOH → Cl ^•^ + H _2_O	1.0 × 10 ^2^ s ^−1^	[ 14]
62	HClOH + Cl ^−^ → Cl _2_ ^•−^ + H _2_O	5.0 × 10 ^9^ M ^−1^s ^−1^	[ 14]
63	Cl _3_ ^−^ + HO _2_ ^•^ → Cl _2_ ^•−^ + HCl + O _2_	1.0 × 10 ^9^ M ^−1^s ^−1^	[ 14]
64	Cl _3_ ^−^ + O _2_ ^•−^ → Cl _2_ ^•−^ + Cl ^−^ + O _2_	3.8 × 10 ^9^ M ^−1^s ^−1^	[ 14]
65	Cl _3_ ^−^ → Cl _2_ + Cl ^−^	1.1 × 10 ^5^ s ^−1^	[ 14]
66	Cl _2_ + O _2_ ^•−^ → Cl _2_ ^•−^ + O _2_	1.0 × 10 ^9^ M ^−1^s ^−1^	[ 14]
67	Cl _2_ + HO _2_ ^•^ → Cl _2_ ^•−^ + H ^+^ + O _2_	1.0 × 10 ^9^ M ^−1^s ^−1^	[ 13]
68	Cl _2_ + H _2_O → Cl _2_OH ^−^ + H ^+^	1.5 × 10 ^1^ M ^−1^s ^−1^	[ 14]
69	Cl _2_ + H _2_O → HOCl + H ^+^ + Cl ^−^	1.0 × 10 ^−3^ M ^−1^s ^−1^	[ 14]
70	Cl _2_ + H _2_O _2_ → 2HCl + O _2_	1.3 × 10 ^4^ M ^−1^s ^−1^	[ 14]
71	Cl _2_OH ^−^ + H ^+^ → Cl _2_ + H _2_O	2.0 × 10 ^10^ M ^−1^s ^−1^	[ 14]
72	Cl _2_OH ^−^ → HOCl + Cl ^−^	6.1 × 10 ^9^ s ^−1^	[ 14]
73	HOCl + ^•^OH → ClO ^•^ + H _2_O	2.0 × 10 ^9^ M ^−1^s ^−1^	[ 13]
74	HOCl + O _2_ ^•−^ → Cl ^•^ + OH ^−^ + O _2_	7.5 × 10 ^6^ M ^−1^s ^−1^	[ 13]
75	HOCl + HO _2_ ^•^ → Cl ^•^ + H _2_O + O _2_	7.5 × 10 ^6^ M ^−1^s ^−1^	[ 14]
76	HOCl + H _2_O _2_ → HCl + H _2_O + O _2_	1.1 × 10 ^4^ M ^−1^s ^−1^	[ 14]
77	ClO ^−^ + ^•^OH → ClO ^•^ + OH ^−^	8.8 × 10 ^9^ M ^−1^s ^−1^	[ 14]
78	ClO ^−^ + H _2_O _2_ → Cl ^−^ + H _2_O + O _2_	1.7 × 10 ^5^ M ^−1^s ^−1^	[ 14]
79	ClO ^−^ + O _2_ ^•−^ + H _2_O → Cl ^•^ + 2OH ^−^ + O _2_	2.0 × 10 ^8^ M ^−1^s ^−1^	[ 14]
80	Cl _2_ ^•−^ + HO _2_ ^•−^ → 2Cl ^−^ + H ^+^ + O _2_	3.0 × 10 ^9^ M ^−1^s ^−1^	[ 14]
**CO** **_3_** **^2−^** ** reactions**			
81	CO _3_ ^2−^ + H ^+^ → HCO _3_ ^−^	5 × 10 ^10^ M ^−1^s ^−1^	[ 21]
82	CO _3_ ^2−^ + ^•^OH → CO _3_ ^•−^ + OH ^−^	3.9 × 10 ^8^ M ^−1^s ^−1^	[ 15]
83	CO _3_ ^2−^ + SO _4_ ^•−^ → CO _3_ ^•−^ + SO _4_ ^2−^	6.1 × 10 ^6^ M ^−1^s ^−1^	[ 22]
84	CO _3_ ^2−^ + Cl ^•^ → CO _3_ ^•−^ + Cl ^−^	5 × 10 ^8^ M ^−1^s ^−1^	[ 23]
85	CO _3_ ^2−^ + Cl _2_ ^•−^ → CO _3_ ^•−^ + 2Cl ^−^	1.6 × 10 ^8^ M ^−1^s ^−1^	[ 21]
86	CO _3_ ^2−^ + ClO ^•^ → CO _3_ ^•−^ + ClO ^−^	6 × 10 ^2^ M ^−1^s ^−1^	[ 24]
87	HCO _3_ ^−^ + H ^+^ → H _2_CO _3_	5 × 10 ^10^ M ^−1^s ^−1^	[ 25]
88	HCO _3_ ^−^ + ^•^OH → CO _3_ ^•−^ + H _2_O	8.6 × 10 ^6^ M ^−1^s ^−1^	[ 15]
89	HCO _3_ ^−^ + Cl ^•^ → CO _3_ ^•−^ + HCl	2.2 × 10 ^6^ M ^−1^s ^−1^	[ 23]
90	HCO _3_ ^−^ + Cl _2_ ^•−^ → 2Cl ^−^ + H ^+^ + CO _3_ ^•−^	8.0 × 10 ^7^ M ^−1^s ^−1^	[ 21]
91	HCO _3_ ^−^ + SO _4_ ^•−^ → CO _3_ ^•−^ + SO _4_ ^2−^ + H ^+^	9.1 × 10 ^6^ M ^−1^s ^−1^	[ 26]
92	H _2_CO _3_ + ^•^OH → CO _3_ ^•−^ + H _2_O + H ^+^	1.0 × 10 ^6^ M ^−1^s ^−1^	[ 14]
93	H _2_CO _3_ → HCO _3_ ^−^ + H ^+^	2.5 × 10 ^4^ M ^−1^s ^−1^	[ 27]
94	CO _3_ ^•−^ + H _2_O _2_ → HCO _3_ ^−^ + HO _2_ ^•^	4.3 × 10 ^5^ M ^−1^s ^−1^	[ 28]
95	CO _3_ ^•−^ + HO _2_ ^−^ → CO _3_ ^2−^ + HO _2_ ^•^	3 × 10 ^7^ M ^−1^s ^−1^	[ 28]
96	CO _3_ ^•−^ + ^•^OH → pro	3 × 10 ^9^ M ^−1^s ^−1^	[ 29]
97	CO _3_ ^•−^ + CO _3_ ^•−^ → pro	3 × 10 ^7^ M ^−1^s ^−1^	[ 29]
98	CO _3_ ^•−^ + O _2_ ^•−^ → CO _3_ ^2−^ + O _2_	6 × 10 ^8^ M ^−1^s ^−1^	[ 29]
99	CO _3_ ^•−^ + 2Br ^−^ → CO _3_ ^2−^ + Br _2_ ^−^	3.4 × 10 ^4^ M ^−1^s ^−1^	[ 24]
100	CO _3_ ^•−^ + ClO ^−^ → CO _3_ ^2−^ + ClO ^•^	5.1 × 10 ^5^ M ^−1^s ^−1^	[ 30]
**NOM reactions**			
101	NOM + HO ^•^ → X	2.5 × 10 ^4^ (mg L ^−1^) ^−1^ s ^−1^	[ 31]
102	NOM + SO _4_ ^•−^ → X	5.1 × 10 ^3^ (mg L ^−1^) ^−1^ s ^−1^	[ 25]

References1

StandardsBT

Determination of cartap hydrochloride content (spectrophotometric method) copyrighted by Bureau of Indian Standards
IS
14159
1994
2

LeeSJ
CaboniP
TomizawaM
CasidaJE

Cartap Hydrolysis Relative to Its Action at the Insect Nicotinic Channel
Journal of Agricultural and Food Chemistry
2004
52
1
95
98
10.1021/jf0306340
147090193

CostaTD
SantosJ
SilvaD
Martinez-HuitleC

BDD-Electrolysis of Oxalic Acid in Diluted Acidic Solutions
Journal of the Brazilian Chemical Society
2019
30
7
1541
1547
10.21577/0103-5053.20190051
4

DávilaOO
BergeronLL
GutiérrezPR
JiménezMMD
SirésI
BrillasE
NavarroAFR
ArandesJB
LlopisJVS

Electrochemical oxidation of dibenzothiophene compounds on BDD electrode in acetonitrile-water medium
Journal of Electroanalytical Chemistry
2019
847
113172
10.1016/j.jelechem.2019.05.054
5

KisacikI
StefanovaA
ErnstS
BaltruschatH

Oxidation of carbon monoxide, hydrogen peroxide and water at a boron doped diamond electrode: the competition for hydroxyl radicals,”
Physical Chemistry Chemical Physics
2013
15
13
4616
10.1039/c3cp44643c
234229496

MitrokaS
ZimmeckS
TroyaD
TankoJM

How Solvent Modulates Hydroxyl Radical Reactivity in Hydrogen Atom Abstractions
Journal of the American Chemical Society
2010
132
9
2907
2913
10.1021/ja903856t
201464697

PupoMM
OlivaJMA
BarriosEKI
Salazar-BandaGR
RadjenovicJ

Characterization and comparison of Ti/TiO2-NT/SnO2–SbBi, Ti/SnO2–SbBi and BDD anode for the removal of persistent iodinated contrast media (ICM)
Chemosphere
2020
253
126701
10.1016/j.chemosphere.2020.126701
323029028

Pacheco-ÁlvarezMO

Improvement of the Degradation of Methyl Orange Using a TiO2/BDD Composite Electrode to Promote Electrochemical and Photoelectro-Oxidation Processes
International Journal of Electrochemical Science
2018
13
11549
11567
10.20964/2018.12.70
9

Martínez-HuitleCA
FerroS
ReynaS
Cerro-LópezM
De BattistiA


Electrochemical oxidation of oxalic acid in the presence of halides at boron doped diamond electrode,”
Journal of the Brazilian Chemical Society
2008
19
1
150
156
10.1590/S0103-50532008000100021
10

YavuzY
ShahbaziR

Anodic oxidation of Reactive Black 5 dye using boron doped diamond anodes in a bipolar trickle tower reactor
Separation and Purification Technology
2012
85
130
136
10.1016/j.seppur.2011.10.001
11

HoangNT
NguyenXC
LePC
JuzsakovaT
ChangSW


Electrochemical degradation of pesticide Padan 95SP by boron-doped diamond electrodes: The role of operating parameters
Journal of Environmental Chemical Engineering
2021
9
3
105205
10.1016/j.jece.2021.105205
12

PanizzaM
KapalkaA
ComninellisC

Oxidation of organic pollutants on BDD anodes using modulated current electrolysis
Electrochimica Acta
2008
53
5
2289
2295
10.1016/j.electacta.2007.09.044
13

GuanYH
MaJ
LiXC
FangJY
ChenLW

Influence of pH on the Formation of Sulfate and Hydroxyl Radicals in the UV/Peroxymonosulfate System
Environmental Science & Technology
2011
45
21
9308
9314
10.1021/es2017363
2199935714

GrebelJE
PignatelloJJ
MitchWA

Effect of Halide Ions and Carbonates on Organic Contaminant Degradation by Hydroxyl Radical-Based Advanced Oxidation Processes in Saline Waters,”
Environmental Science & Technology
2010
44
17
6822
6828
10.1021/es1010225
2068156715

BuxtonGV
GreenstockCL
HelmanWP
RossAB

Critical Review of rate constants for reactions of hydrated electrons, hydrogen atoms and hydroxyl radicals (•OH/•O − in Aqueous Solution
Journal of Physical and Chemical Reference Data
1988
17
2
513
886
10.1063/1.555805
16

LiangC
WangZS
MohantyN

Influences of carbonate and chloride ions on persulfate oxidation of trichloroethylene at 20 °C
Science of The Total Environment
2006
370
2–3
271
277
10.1016/j.scitotenv.2006.08.028
1701489117

WeinsteinJ
BielskiBHJ

Kinetics of the interaction of perhydroxyl and superoxide radicals with hydrogen peroxide. The Haber-Weiss reaction
Journal of the American Chemical Society
1979
101
1
58
62
10.1021/ja00495a010
18

YuXY
BaoZC
BarkerJR

Free Radical Reactions Involving Cl•, Cl2•-, and SO4•- in the 248 nm Photolysis of Aqueous Solutions Containing S2O82- and Cl-
The Journal of Physical Chemistry A
2004
108
2
295
308
10.1021/jp036211i
19

MaruthamuthuP
NetaP

Radiolytic chain decomposition of peroxomonophosphoric and peroxomonosulfuric acids
The Journal of Physical Chemistry
1977
81
10
937
940
10.1021/j100525a001
20

DasTN

Reactivity and Role of SO5•- Radical in Aqueous Medium Chain Oxidation of Sulfite to Sulfate and Atmospheric Sulfuric Acid Generation
The Journal of Physical Chemistry A
2001
105
40
9142
9155
10.1021/jp011255h
21

MatthewBM
AnastasioC

A chemical probe technique for the determination of reactive halogen species in aqueous solution: Part 1 – bromide solutions
Atmospheric Chemistry and Physics
2006
6
9
2423
2437
10.5194/acp-6-2423-2006
22

ZuoZ
CaiZ
KatsumuraY
ChitoseN
MuroyaY

Reinvestigation of the acid–base equilibrium of the (bi)carbonate radical and pH dependence of its reactivity with inorganic reactants
Radiation Physics and Chemistry
1999
55
1
15
23
10.1016/S0969-806X(98)00308-9
23

MertensR
SonntagCV

Photolysis (λ = 354 nm of tetrachloroethene in aqueous solutions
Journal of Photochemistry and Photobiology A: Chemistry
1995
85
1–2
1
9
10.1016/1010-6030(94)03903-8
24

HuieRE
CliftonCL
NetaP

Electron transfer reaction rates and equilibria of the carbonate and sulfate radical anions
Int J Radiat Appl Instrumentation. Part C Radiation Physics and Chemistry
1991
38
5
477
481
10.1016/1359-0197(91)90065-A
25

YangY
PignatelloJJ
MaJ
MitchWA

Comparison of Halide Impacts on the Efficiency of Contaminant Degradation by Sulfate and Hydroxyl Radical-Based Advanced Oxidation Processes (AOPs)
Environmental Science & Technology
2014
48
4
2344
2351
10.1021/es404118q
2447938026

DogliottiL
HayonE

Flash photolysis of per[oxydi]sulfate ions in aqueous solutions. The sulfate and ozonide radical anions
The Journal of Physical Chemistry
1967
71
8
2511
2516
10.1021/j100867a019
27

SunkaP
BabickýV
ClupekM
LukesP
SimekM


Generation of chemically active species by electrical discharges in water
Plasma Sources Science and Technology
1999
8
258
258
10.1088/0963-0252/8/2/006
28

DraganićZD
Negrón-MendozaA
SehestedK
VujoševićSI
Navarro-GonzálesR


Radiolysis of aqueous solutions of ammonium bicarbonate over a large dose range
International Journal of Radiation Applications and Instrumentation. Part C Radiation Physics and Chemistry
1991
38
3
317
321
10.1016/1359-0197(91)90100-G
29

CrittendenJC
HuS
HandDW
GreenSA

A kinetic model for H2O2/UV process in a completely mixed batch reactor
Water Research
1999
33
10
2315
2328
10.1016/S0043-1354(98)00448-5
30

AlfassiZB
HuieRE
MosseriS
NetaP

Kinetics of one-electron oxidation by the ClO radical,”
Int J Radiat Appl Instrumentation. Part C Radiation Physics and Chemistry
1988
32
1
85
88
10.1016/1359-0197(88)90018-5
31

WuaZ
FangJ
XiangY
ShangC
LiX


Roles of reactive chlorine species in trimethoprim degradation in the UV/chlorine process: Kinetics and transformation pathways
Water Research
2016
104
272
282
10.1016/j.watres.2016.08.011
2754434932

ColedamDAC
AquinoJM
SilvaBF
SilvaAJ
Rocha-FilhoRC

Electrochemical mineralization of norfloxacin using distinct boron-doped diamond anodes in a filter-press reactor, with investigations of toxicity and oxidation by-products
Electrochimica Acta
2016
213
856
864
10.1016/j.electacta.2016.08.003
33

ZajdaM
AleksanderKU

Wastewater treatment methods for effluents from the confectionery industry - an overview
Journal of Ecological Engineering
2019
20
9
293
304
10.12911/22998993/112557
34

DongH
ZengG
TangL
FanC
ZhangC


An overview on limitations of TiO_2_-based particles for photocatalytic degradation of organic pollutants and the corresponding countermeasures
Water Research
2015
79
128
146
10.1016/j.watres.2015.04.038
2598091435

Jojoa-SierraSD
Silva-AgredoJ
Herrera-CalderonJE
Torres-PalmaRA

Elimination of the antibiotic norfloxacin in municipal wastewater, urine and seawater by electrochemical oxidation on IrO2 anodes
Science of the Total Environment
2016
575
1228
1238
10.1016/j.scitotenv.2016.09.201
2772025136

PanizzaM
CerisolaG

direct and mediated anodic oxidation of organic pollutants
Chemical Reviews
2009
109
12
6541
6569
10.1021/cr9001319
1965840137

HoangNT
HolzeR

Degradation of pesticide Cartap in Padan 95SP by combined advanced oxidation and electro-Fenton process
Journal of solid state electrochemistry
2021
25
173
184
10.1007/s10008-020-04581-7
38

HoangNT

Physical and electrochemical properties of Boron-Doped Diamond (BDD) electrode
Journal of Science and Technology
2020
18
6
41
45
39

MoraoA
LopesA
PessoadeamorimM
GoncalvesI

Degradation of mixtures of phenols using boron doped diamond electrodes for wastewater treatment
Electrochimica Acta
2004
49
9–10
1587
1595
10.1016/S0013-4686(03)00966-6
40
Standards BI
Determination of cartap hydrochloride content (spectrophotometric method) copyrighted by Bureau of Indian Standards,”
IS
141591994
41

LeeSJ
CaboniP
TomizawaM
CasidaJE

Cartap hydrolysis relative to its action at the insect nicotinic channel
Journal of Agricultural and Food Chemistry
2004
52
1
95
98
10.1021/jf0306340
1470901942

ZhangF
SunZ
CuiJ

Research on the mechanism and reaction conditions of electrochemical preparation of persulfate in a split-cell reactor using BDD anode
RSC Advances
2020
10
56
33928
33936
10.1039/D0RA04669H
35519076PMC905671543

WangHW
BringansC
HickeyAJR
WindsorJA
KilmartinP


Cyclic Voltammetry in Biological Samples: A Systematic Review of Methods and Techniques Applicable to Clinical Settings
Signals
2021
2
1
138
158
10.3390/signals2010012
44

LiuZ
DingH
ZhaoC
WangT
WangP


Electrochemical activation of peroxymonosulfate with ACF cathode: Kinetics, influencing factors, mechanism, and application potential
Water Research
2019
159
111
121
10.1016/j.watres.2019.04.052
3108264245

HoangNT
NguyenXC
LePC
JuzsakovaT
ChangSW


Electrochemical degradation of pesticide Padan 95SP by boron-doped diamond electrodes: The role of operating parameters
Journal of Environmental Chemical Engineering
2021
9
3
105205
10.1016/j.jece.2021.105205
46

LeiY
LuJ
ZhuM
XieJ
PengS


Radical chemistry of diethyl phthalate oxidation via UV/peroxymonosulfate process: Roles of primary and secondary radicals
Chemical Engineering Journal
2020
379
122339
10.1016/j.cej.2019.122339
47

LianL
YaoB
HouS
FangJ
YanS


Kinetic study of hydroxyl and sulfate radical-mediated oxidation of pharmaceuticals in wastewater effluents
Environmental Science & Technology
2017
51
5
2954
2962
10.1021/acs.est.6b05536
2815165248

LiW
JainT
IshidaK
LiuH

A mechanistic understanding of the degradation of trace organic contaminants by UV/hydrogen peroxide, UV/persulfate and UV/free chlorine for water reuse
Environmental Science: Water Research & Technology
2017
3
1
128
138
10.1039/C6EW00242K
49

HoangNT
NguyenVT
TuanNDM
ManhTD
LePC


Degradation of dyes by UV/Persulfate and comparison with other UV-based advanced oxidation processes: Kinetics and role of radicals
Chemosphere
2022
298
134197
10.1016/j.chemosphere.2022.134197
3527611150

CaiJ
ZhouM
PanY
DuX
LuX

Extremely efficient electrochemical degradation of organic pollutants with co-generation of hydroxyl and sulfate radicals on Blue-TiO2 nanotubes anode
Applied Catalysis B: Environmental
2019
257
117902
10.1016/j.apcatb.2019.117902
51

JiY
ShiY
WangL
LuJ

Denitration and renitration processes in sulfate radical-mediated degradation of nitrobenzene
Chemical Engineering Journal
2017
315
591
597
10.1016/j.cej.2017.01.071
52

BuxtonGV
GreenstockCL
HelmanWP
RossAB

Critical Review of rate constants for reactions of hydrated electrons, hydrogen atoms and hydroxyl radicals (•OH/•O − in Aqueous Solution
Journal of Physical and Chemical Reference Data
1988
17
2
513
886
10.1063/1.555805
53

NetaP
HuieRE
RossAB

Rate Constants for reactions of inorganic radicals in aqueous solution
Journal of Physical and Chemical Reference Data
1988
17
3
1027
1284
10.1063/1.555808
54

TanC
JianX
WuH
ShengT
SunTK


Kinetics degradation of phenacetin by solar activated persulfate system
Separation and Purification Technology
2021
256
117851
10.1016/j.seppur.2020.117851
55

SerranoK
MichaudPA
ComninellisC
SavallA

Electrochemical preparation of peroxodisulfuric acid using boron doped diamond thin film electrodes
Electrochimica Acta
2002
48
4
431
436
10.1016/S0013-4686(02)00688-6
56

AhnaYY
BaeH
KimHI
KimSH
KimJH


Surface-loaded metal nanoparticles for peroxymonosulfate activation: Efficiency and mechanism reconnaissance
Applied Catalysis B: Environmental
2019
241
561
569
10.1016/j.apcatb.2018.09.056
57

DavisJ
BaygentsJC
FarrellJ

Understanding persulfate production at boron doped diamond film anodes
Electrochimica Acta
2014
150
68
74
10.1016/j.electacta.2014.10.104
58

KhamisD
MahéE
DardoizeF
DevilliersD

Peroxodisulfate generation on boron-doped diamond microelectrodes array and detection by scanning electrochemical microscopy
Journal of Applied Electrochemistry
2010
40
10
1829
1838
10.1007/s10800-010-0114-x


## Figures and Tables

**Table.** The overview of the contribution of oxidants to CT degradation.

	DET	•OH	${\text{SO}}_{4}^{•-}$	other ROS
*k_CT_* by oxidant, min^−1^	0.007	0.024	0.005	0.003
Contribution, %	15	61.5	12.8	8.5
Concentration	-	3.2 × 10^−13^ M	5.8 × 10^−14^ M	(no detection)
