# Supplementary figures and images for: Insight into electrochemical degradation of Cartap (in Padan 95SP) by boron-doped diamond electrode: kinetic and effect of water matrices
Source: Turk J Chem. 2022 May 11;46(5):1733–43. doi: 10.55730/1300-0527.3476 (PMC10390180; doi:10.55730/1300-0527.3476)

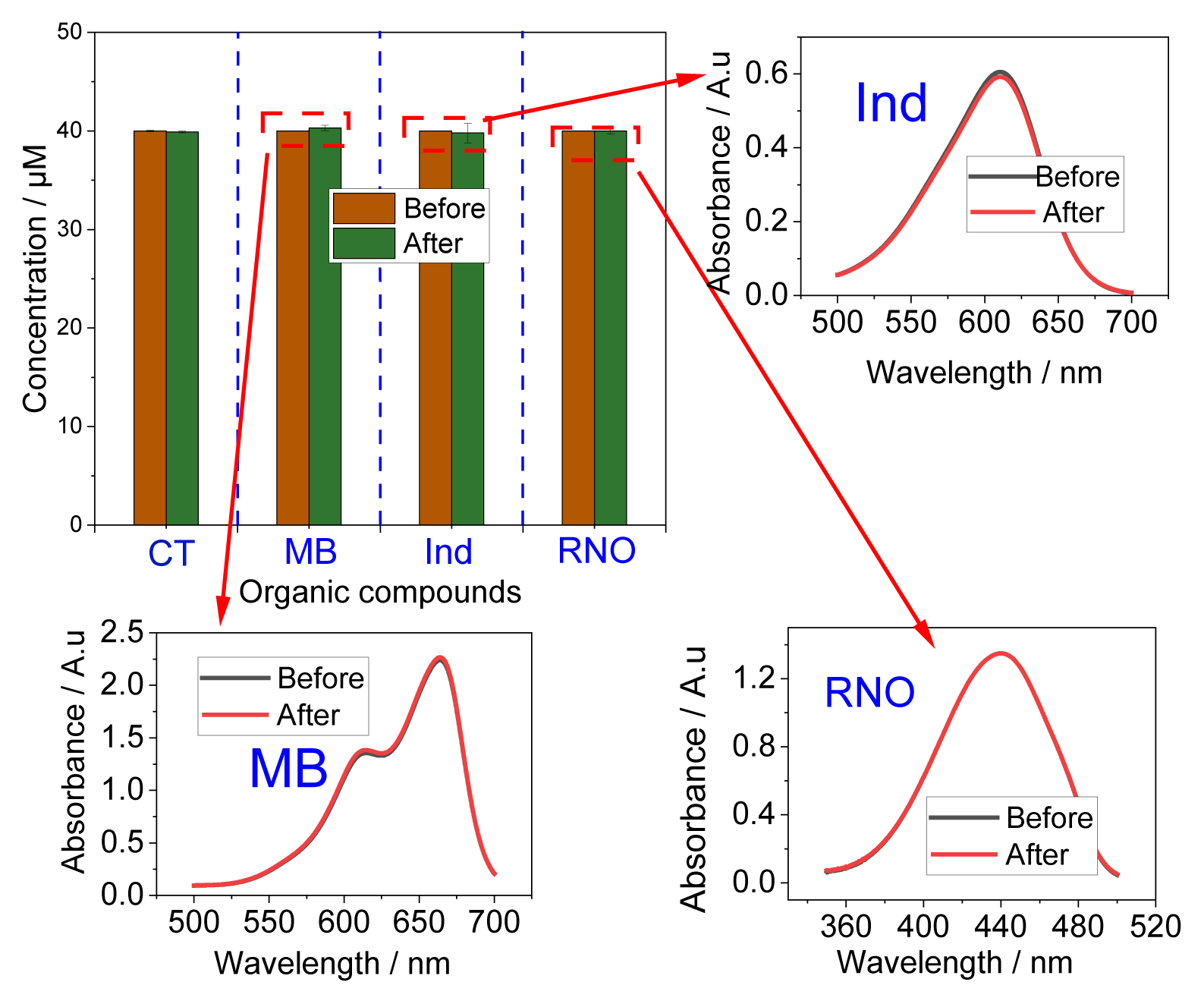

Supplement: Figure S1. — Concentration of organic compounds before and after immersed BDD electrode: CT (Cartap), MB (methylene blue), Ind (indigo carmine), RNO (P-nitrosodimethylaniline). The initial concentration of organic compounds was 40 μM, Vsolution = 100 mL. [file turkjchem-46-5-1733s1.tif]

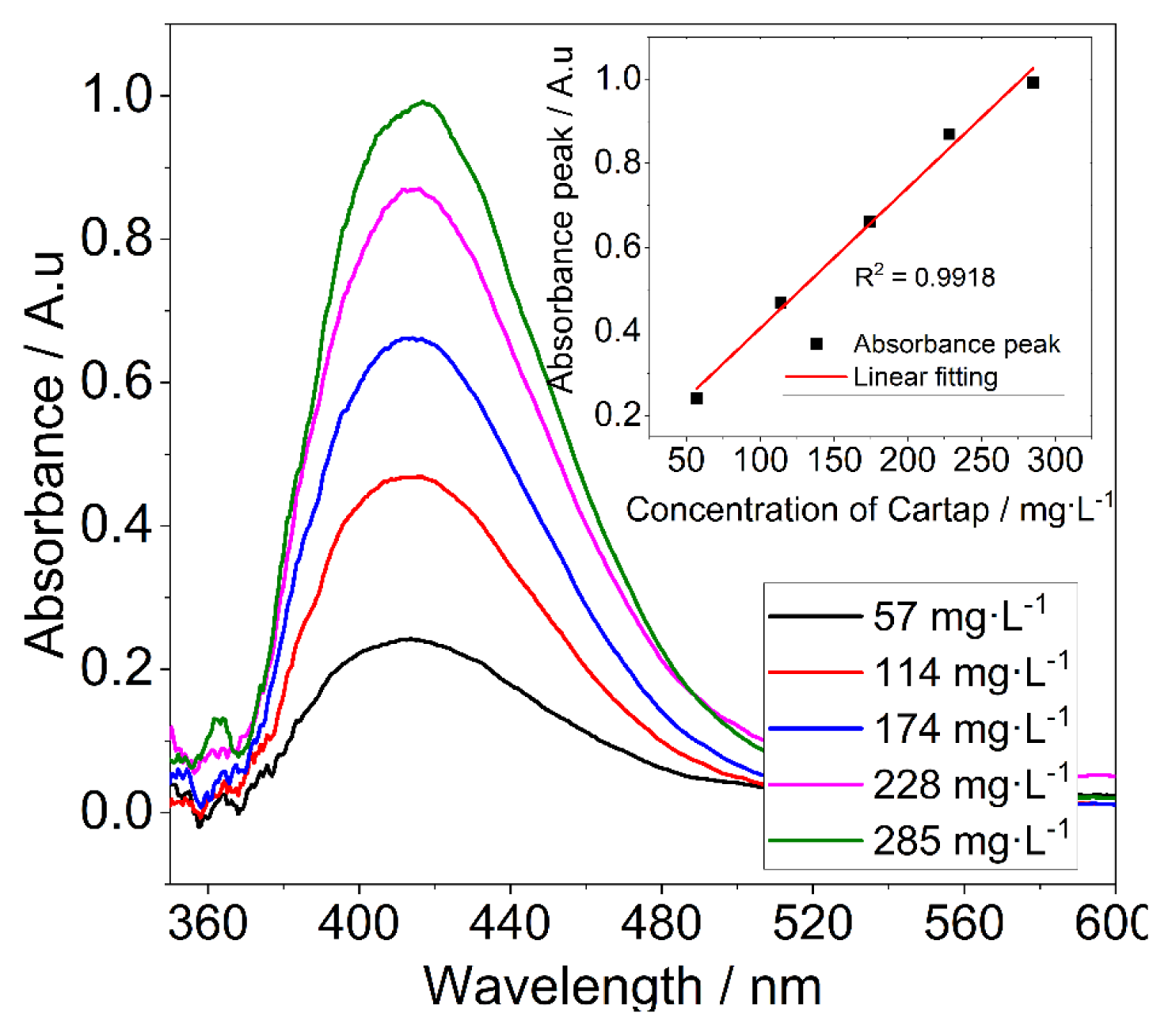

Supplement: Figure S2. — The intensity of absorbance spectra for calculating CT concentration in water. Insert: Calibration plot for calculating CT concentration based on data of Figure 2. [file turkjchem-46-5-1733s2.tif]

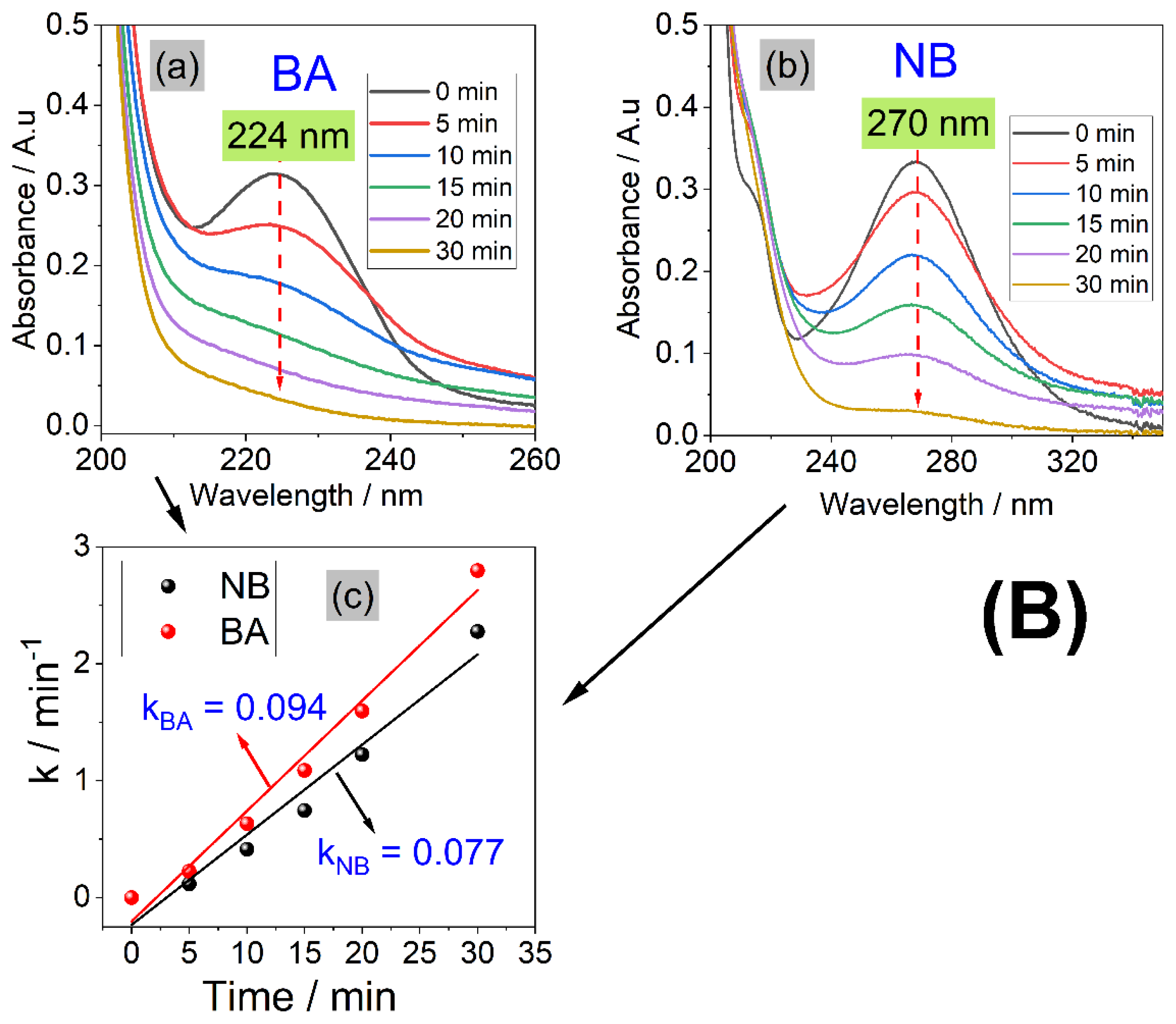

Supplement: Figure S3 — The kinetic degradation of NB and BA (by HPLC detection (A) and by UV-Vis detection (B)) to determine the concentration of •OH and SO4•-. Experimental conditions: [NB] = [BA] = 40 μM, [Na2SO4] = 0.05 M, pH = 3, V = 250 mL, current density j = 40 mA cm−2. [file turkjchem-46-5-1733s3.tif]

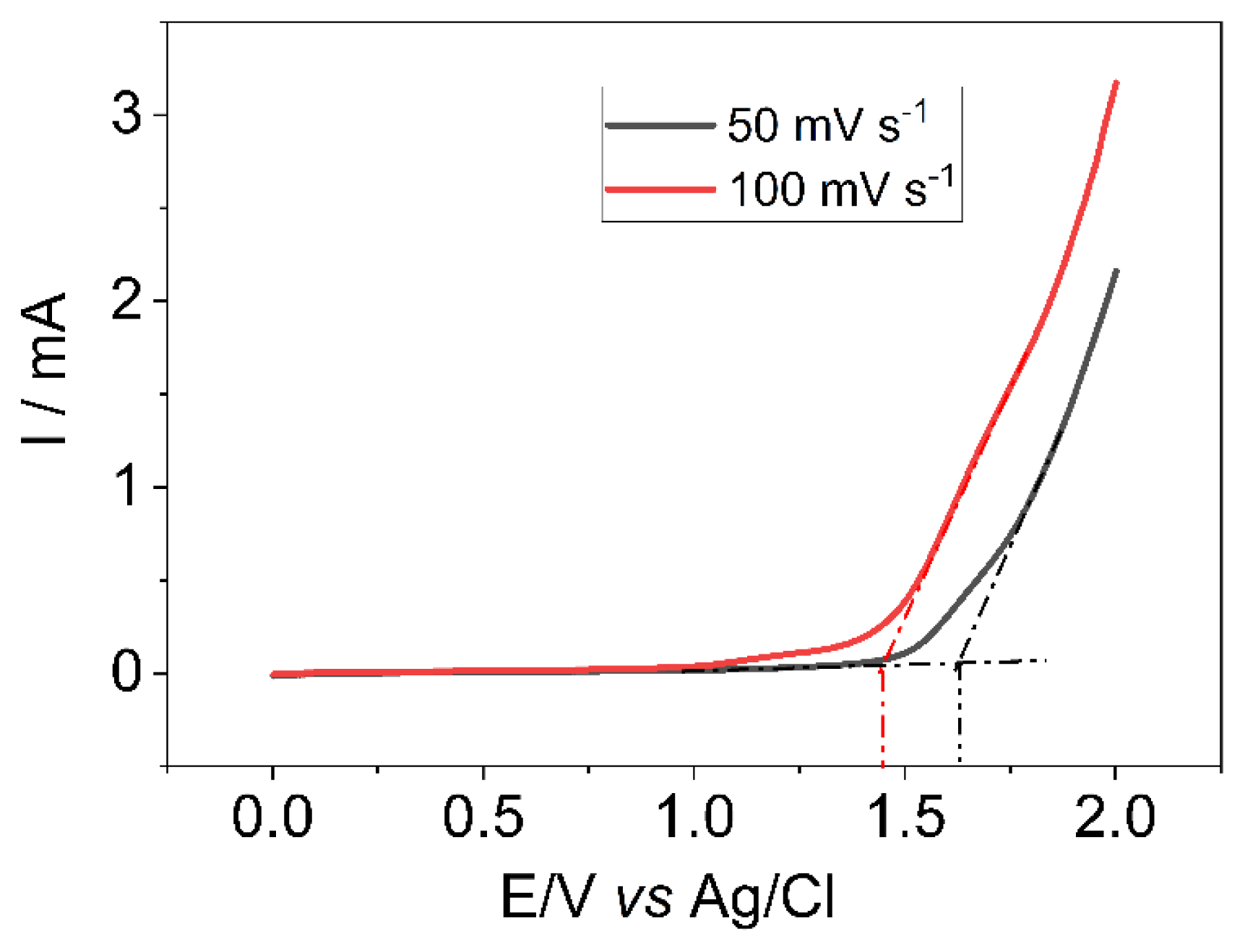

Supplement: Figure S4. — LSV of BDD in 0.05 M Na2SO4 at two scan rates. pH = 6.5 and at room temperature. [file turkjchem-46-5-1733s4.tif]

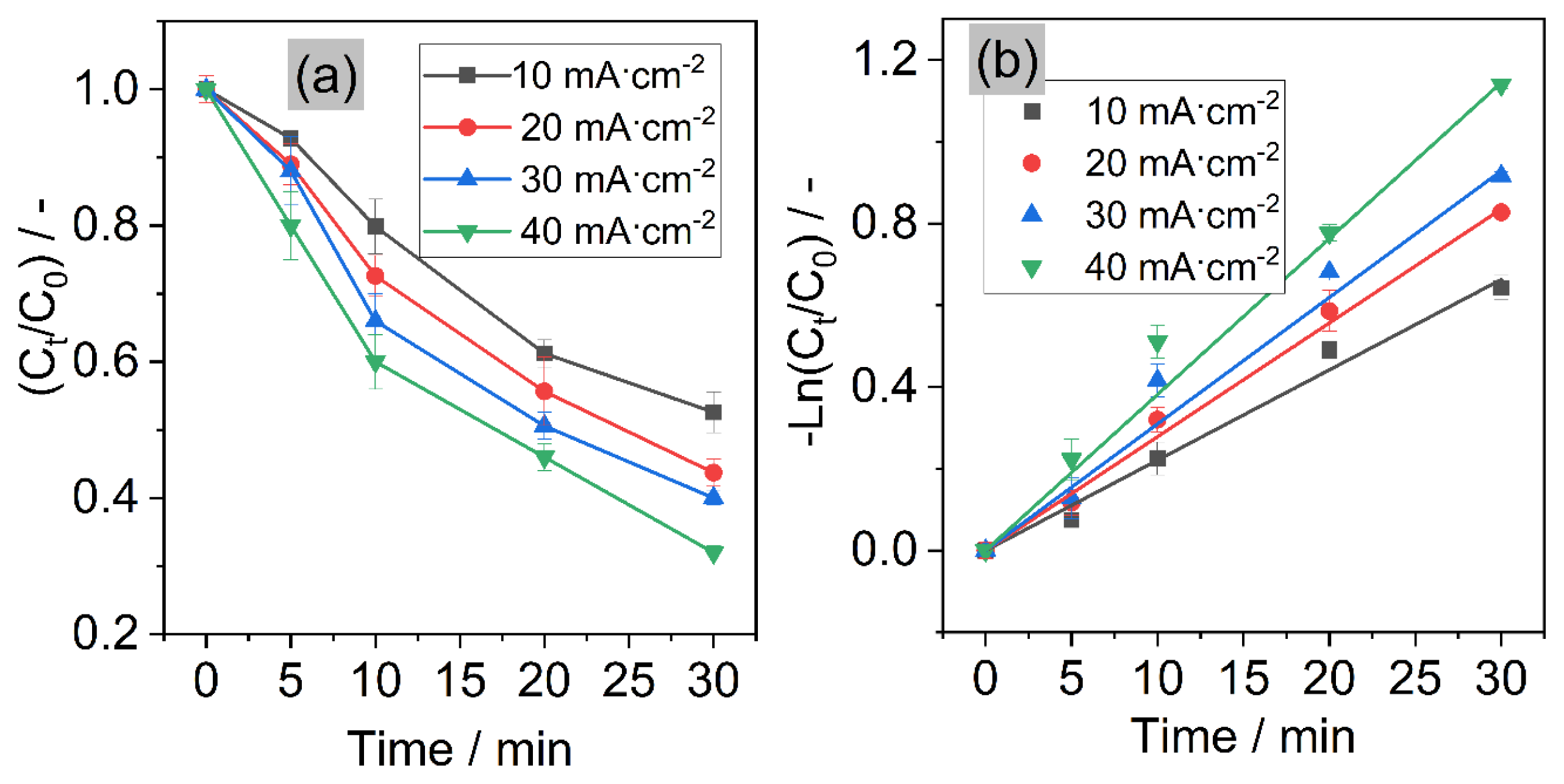

Supplement: Figure S5 — a) Effect of j on CT degradation as a function of time (j from 10 to 40 mA cm−2). b) Effect of j on the apparent rate constant. Initial concentration of Padan 95SP (95% CT): 40 μM, supporting electrolyte: 0.05 M Na2SO4, pH = 3, BDD: working electrode. [file turkjchem-46-5-1733s5.tif]

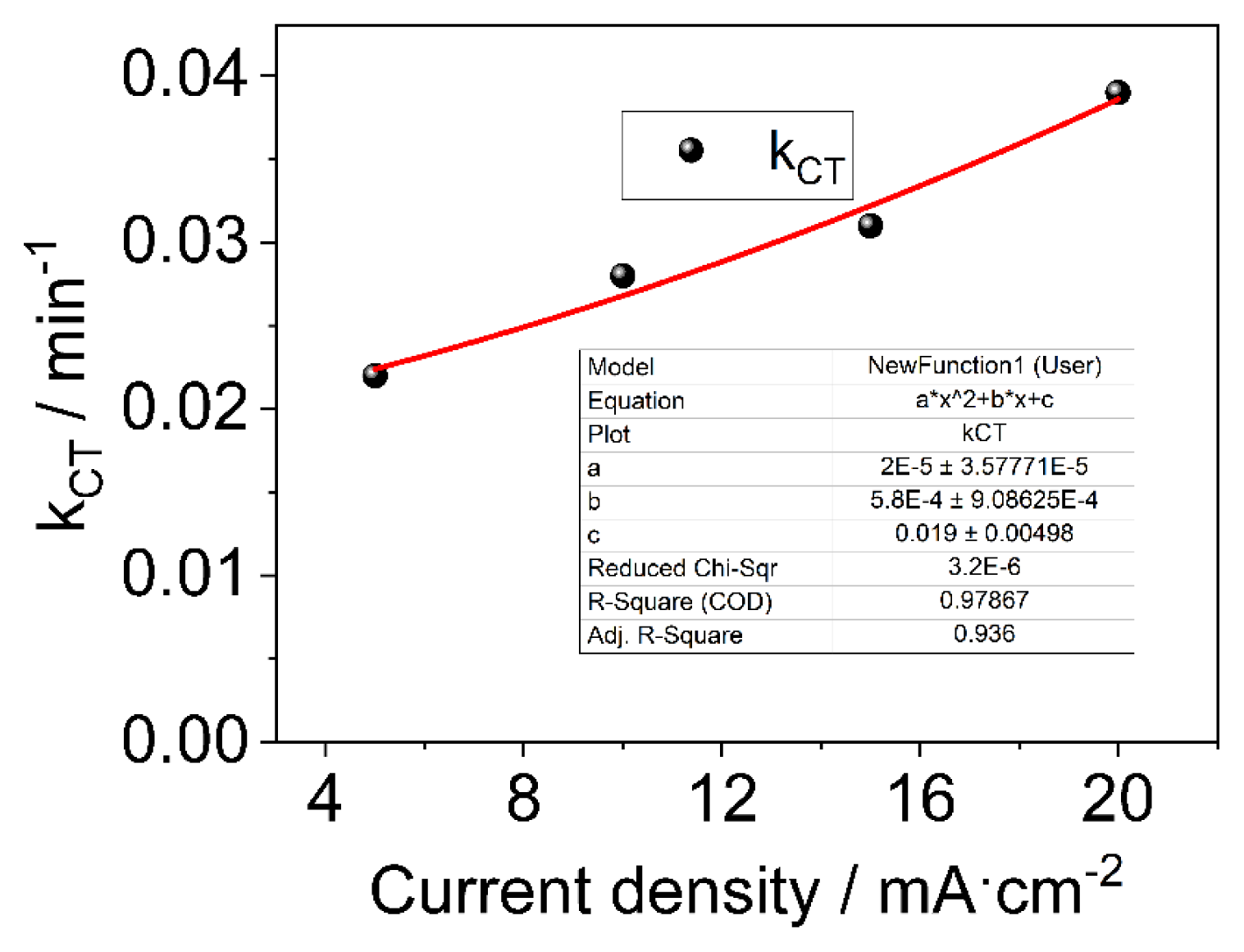

Supplement: Figure S6. — The plot of degradation rate constant versus current density. [file turkjchem-46-5-1733s6.tif]
